# Two steps are better than one: improving gene editing to treat cancer

**DOI:** 10.1002/2211-5463.13342

**Published:** 2021-12-26

**Authors:** Francesca Minter

**Affiliations:** ^1^ FEBS Open Bio Editorial Office Cambridge UK

## Abstract

Gene editing enables scientists to make precise changes to the genome of an organism using the cell’s own ability to repair damaged DNA using a supplied DNA template. In recent years, gene editing has been applied clinically in the treatment of diseases such as cancer. Gene editing has been used in a type of immunotherapy, known as chimeric antigen receptor‐expressing T cell (CAR‐T) therapy, to restore the body’s ability to find and kill specific cancer cells. For this therapy, viruses are often used to supply the cell with the DNA template used for creating the edit in the target DNA. However, the use of viruses in this context is laborious and costly. Developing non‐viral methods for delivery of DNA templates for gene editing would circumvent these problems, but current methods can have toxic effects on cells and result in low editing efficiency. In a new article published in this issue, Yang et al. describe a novel method for viral‐independent delivery of naked DNA and its incorporation into the genome for engineering cells for CAR‐T therapy.

## Graphical abstract

Gene editing can be used to restore the body’s ability to kill specific cancer cells. Viruses are often used to supply the cell with the DNA template used for creating the edit in the target DNA, but this is laborious and costly. In this issue, Yang et al. describe a novel method for viral‐independent delivery of naked DNA for engineering cells for CAR‐T therapy.

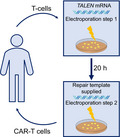

**Abbreviations**
CAR‐T: chimeric antigen receptor‐expressing T cells
TALEN: transcription activator‐like effector nuclease
*TRAC*
: T‐cell receptor alpha constant region

In recent years, advances in research and treatment options have improved the prognosis and life expectancy of many people with cancer. The immune system includes white blood cells, such as T cells, which recognise misfunctioning or infected cells and target them for destruction. However, cancerous cells can evade attack by the immune system, leading to uncontrolled growth and the potential for metastasis into different tissues [1]. Immunotherapy drugs act by restoring the function of immune cells to enable them to recognise and eliminate cancerous cells. Chimeric antigen receptor‐expressing T cell (CAR‐T) therapy is a type of gene editing‐based immunotherapy that involves engineering immune cells, specifically T cells, to recognise and destroy cancer cells. This treatment can have profound antitumour effects in patients with blood cancer, dramatically improving survival rates [2].

Gene editing enables scientists to make precise changes to the DNA in a cell. This technology harnesses the cell’s built‐in machinery to repair damage to DNA. Firstly, the cellular DNA is cut by a specialised enzyme called a nuclease. One class of nucleases used for gene editing are the transcription activator‐like effector nucleases (TALENs), which can be engineered to target specific DNA sequences and have been used for gene editing of T cells to generate CAR‐T cells for therapy [3]. The TALENs are delivered as mRNA, which reduces the chance of introducing undesired mutations as it cannot integrate into the cellular DNA. In addition to a nuclease, researchers also need to supply an exogenous DNA sequence which guides the repair process. A viral vector is often used to carry such DNA sequences into the cell. However, there is a limit to the size of DNA that can be carried, and viral vectors can be expensive and laborious to produce [4]. Viral‐free delivery of DNA would circumvent these limitations, but naked DNA can be toxic to cells and methods so far have resulted in limited editing efficiency. As CAR‐T therapy extends to target different cancers, it is important to overcome these limitations to enable rapid and cost‐effective development of new CAR‐T therapies.

In this issue, Yang et al. [4] describe an optimised method for virus‐independent delivery of repair templates for TALEN‐mediated editing of T cells for use in CAR‐T therapy. The authors first introduced (transfected) *TALEN* mRNA into human T cells. At 24 h after transfection, the quantity of *TALEN* mRNA was 31% of the amount at initial transfection. The amount of TALEN protein increased over time until 20 h and was undetectable after 48 h. The authors hypothesised that by introducing a repair template at the peak of TALEN protein expression levels (i.e. at 20 h), they could increase the efficiency of gene editing. Rather than introducing *TALEN* (as an mRNA) and the template (a DNA sequence) into the cell at the same time through a one‐step process, they would introduce them at different times in a two‐step process, so that the DNA would be introduced when TALEN protein expression was at its peak (Fig. 1). Moreover, as the delivery of mRNA and DNA do not always involve the same procedure, separating the process into two allows independent optimisation of each step.

**Fig. 1:**
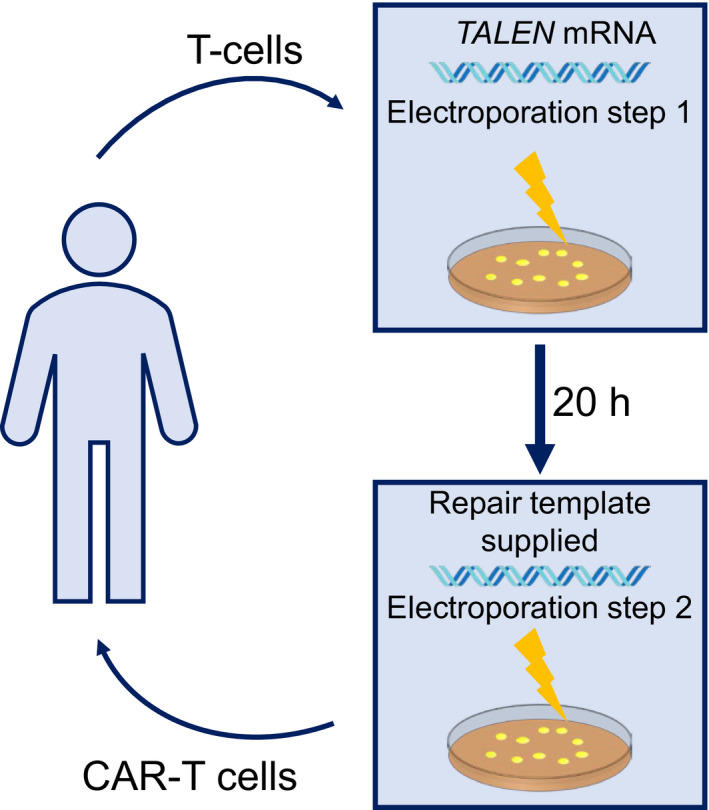
Two‐step electroporation for viral‐independent delivery of template DNA for *ex vivo* gene editing of T cells for CAR‐T therapy. The first electroporation introduces *TALEN* mRNA, and then, 20 h later at peak TALEN protein expression, the second electroporation delivers the repair template DNA to complete gene editing and create viable T cells.

The authors examined the effects on gene editing of introducing the DNA repair template at different times after transfection with *TALEN* mRNA, observing a threefold higher insertion frequency at 16 h as compared to 0 h. Critically, the authors also tested whether the edited cells were viable and could function as immune cells. To do this, they measured the number of viable cells over time and found that there was no statistically significant difference between the number of cells at 0 and 20 h post‐DNA electroporation. Furthermore, the authors found that the T cells which had been engineered using their two‐step electroporation method were better at killing target cancer cells *in vitro*.

Overall, the authors demonstrated that two electroporation steps, the first to introduce *TALEN* mRNA and the second to introduce the repair template at the time of peak TALEN expression, leads to the highest number of edited CAR‐T cells. They also showed that these are viable and functioning immune cells. Although this study focussed on the use of TALENs for gene editing, future work could include testing whether other gene editing technologies, such as CRISPR, produce similar results. The development of methods to reduce the cost and increase the efficiency of gene editing in CAR‐T therapy may facilitate the target and treatment of additional cancer types.

## Conflict of interest

The author declares no conflict of interest.

## Author contributions

FM wrote the article and created the figure.
